# Information Limited Oligonucleotide Amplification Assay for Affinity-Based, Parallel Detection Studies

**DOI:** 10.1371/journal.pone.0151072

**Published:** 2016-03-15

**Authors:** Harish Bokkasam, Albrecht Ott

**Affiliations:** Institute of Biological Experimental Physics, Saarland University, Saarbrucken, Germany; University of North Carolina at Charlotte, UNITED STATES

## Abstract

Molecular communication systems encounter similar constraints as telecommunications. In either case, channel crosstalk at the receiver end will result in information loss that statistical analysis cannot compensate. This is because in any communication channel there is a physical limit to the amount of information that can be transmitted. We present a novel and simple modified end amplification (MEA) technique to generate reduced and defined amounts of specific information in form of short fragments from an oligonucleotide source that also contains unrelated and redundant information. Our method can be a valuable tool to investigate information overflow and channel capacity in biomolecular recognition systems.

## Introduction

Channel capacity corresponds to the highest rate of information transfer that can be achieved in a communications channel. It depends, among other factors, on the signal to noise ratio of the channel. If information is transmitted at a rate above channel capacity, the amount of erroneously received information unavoidably increases and information is lost irreversibly. This is regardless of the statistical treatment that is performed at the receiver, and independent of the coding scheme used. Although there have been many advances in increasing the accuracy of communication systems through novel electronic circuitry, channel capacity still defines the physical limit for accurate information transmission as shown in Shannon’s work on information theory [1]. Information theory has been extended to molecular information processing in Life science [2].

DNA constitutes a four letter code. Molecules that bind to DNA, so called transcription factors, regulate gene expression [3]. It has been suggested that in the biological cell, the number of different transcription factors is such that they reach the physical limit of information density at the DNA recognition sequence [4]. In the biological cell molecular recognition pairs work in parallel and they do not seem to interfere. Understanding their action in an information theoretical context could help and enhance our understanding of biological molecular self organization [5].

**Fig 1:**
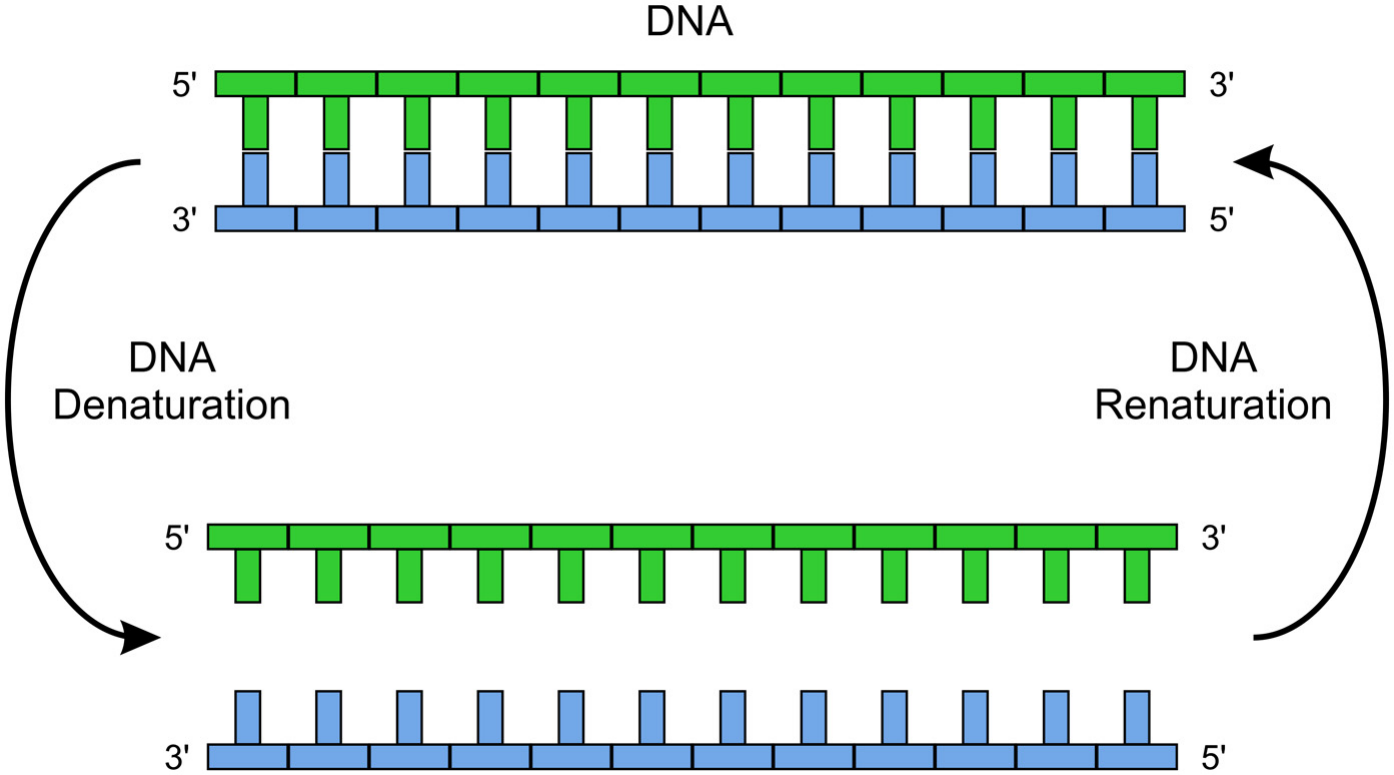
Specific molecular recognition. The figure illustrates the sequence specific temperature dependent nucleic acid hybridization of a DNA. The strand (TGACATGCTAATC) is complementary to ssDNA strand (ACTGTCGATTAG).

DNA single strands can combine (‘hybridize’) to form the double helical structure if their sequences are complementary. Fig 1 illustrates the molecular recognition of oligonucleotide strands. Specificity results from collective action, where binding of one base enhances the probability of the neighboring complementary bases to bind. Although hybridization will tolerate a few defects, it is considered highly specific [6]. Specific nucleic acid hybridization is the working principle for many gene expression profiling techniques [7].

DNA microarrays [8] are a high throughput technique that consists of DNA single strands grafted onto a surface. Complementary target strands in solution hybridize to these strands [9]. Concentrations of these hybridized sequences are quantitatively determined with markers bound to the targets. It is now admitted that DNA microarrays tend to reliably capture the highly concentrated gene products only [10]. We have shown that in simple situations the impact of defects on hybridization binding affinity can be predicted very well [11] and other detailed predictions are possible as well.

Microarray based techniques generally rely on random oligonucleotide fragmentation techniques for large scale information gathering and analysis [12]. This provides large amounts of information fragments from a biological source of interest. Industrial microarrays have steadily increased the number of nucleotide sequences that are immobilized on the surface in the goal of better statistical treatment [13]. Combined with advanced bioinformatics and statistical analysis, huge amounts of data can be obtained and analysed from the biological source.

**Fig 2:**
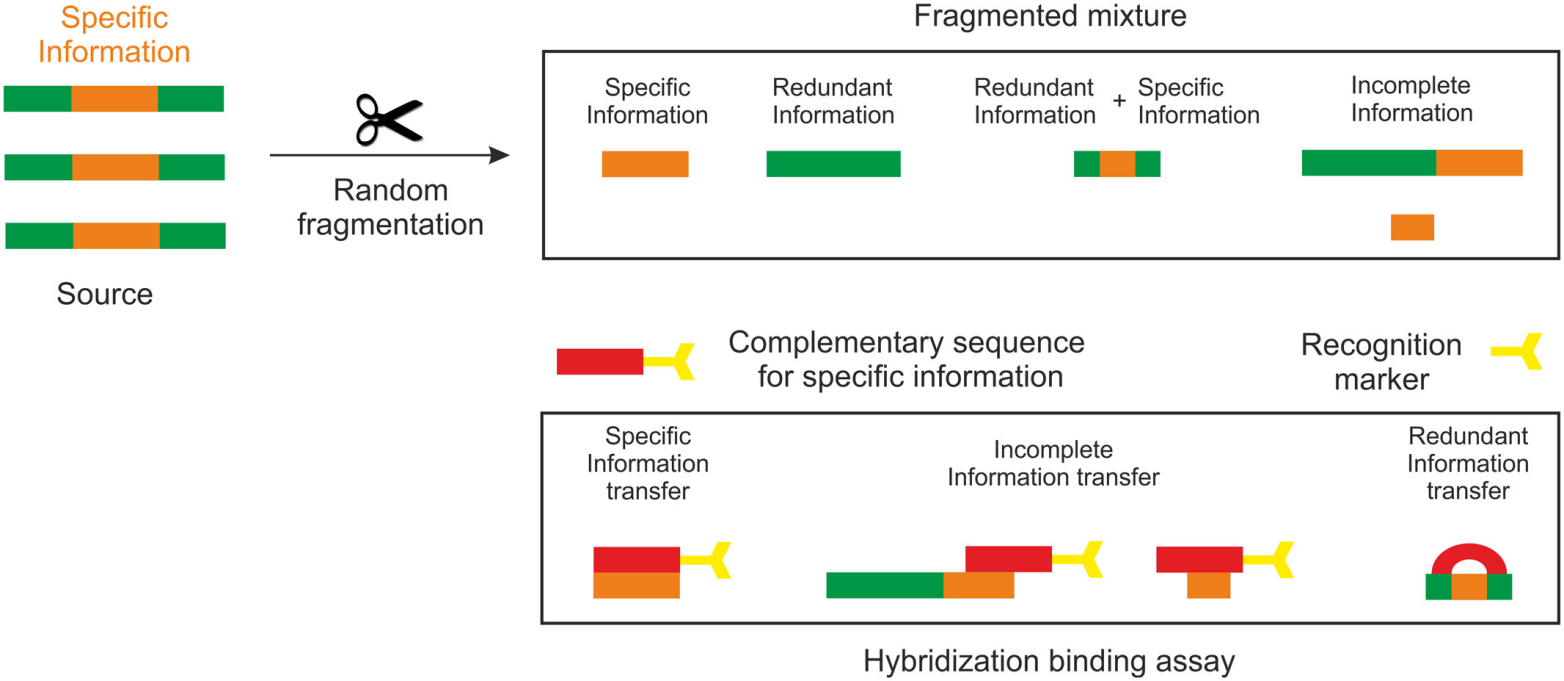
Scheme of a fragmented mixture generated with random fragmentation followed by detection. Specific information is illustrated as an orange band in the middle of a source strand. It is surrounded on both sides by redundant information (green). Specific hybridization corresponds to exact binding of specific information to its complement. Random fragmentation techniques generally generate redundant information along with specific information (Fragmented mixture). This leads to incomplete and/or redundant information transfer in a binding assay.

For DNA microarrays, however, the above means that beyond a certain complexity of the target mixture, due to limited channel capacity, different oligonucleotide molecules must interfere and information is irreversibly lost. We have shown that single strands that hybridize by forming bulged loops bind to single stranded DNA with strong affinity [14]. For random sequences, one can expect binding probabilities between different strands on average to increase exponentially with the length of the strands. In some biological studies only limited amounts of specific information are required [15]. In such a case, random fragmentation techniques will produce unwanted strands that could potentially cloud specific information. Fig 2 shows a cartoon of a fragmented mixture generated with random fragmentation techniques that illustrates problems resulting from unspecific and incomplete information transfer.

To date the physical limit of information that can be transmitted via a binding assay remains poorly understood. Understanding this limit will have impact on the development of high throughput binding assays, and it could play a role to improve our understanding of biological systems. For a study on this subject, it can be advantageous to develop a technique that generates reduced amounts of specific information. In the following we present such a technique, based on DNA. DNA hybridization represents a simple case of molecular recognition, where many techniques and a lot of knowledge already exist.

## Materials and Methods

Primers were from Metabion GmbH. PCR according to protocols provided by Axon Lab. For singleplex and multiplex PCR reactions, final primer concentration was 50–75 nM. Denaturation (95°C), Annealing (50°C) and extension (72°C) were used for PCR amplification. Templates for MEA technique were dsDNA sequences amplified from lambda DNA template. A single primer is used for linear amplifications [16] at the same temperatures. In some experiments the Ladderman DNA Labeling Kit from Takara Bio Europe GmbH was employed. This kit is typically used for primer labeling and primer extension with incorporation of labeled nucleotides. For our purpose, instead of random primers provided with the kit, specific primers were used to generate complementary ssDNA oligonucleotide fragments of predefined lengths. This kit has Bca polymerase, which enables linear isothermal amplification without need for multiple amplification cycles. Primer concentration was 200–300 nM for sufficient ssDNA product required for downstream applications. Three specific primers were used to generate complementary ssDNA fragments of lengths 30, 40 and 50 nt from complex DNA mixture: primer for 30 nt ssDNA product, TATAAATTCTGATTAGCCAG, primer for 40 nt ssDNA product, GTTCGCGGCGGCATTCATCC primer for 50 nt ssDNA product, ACGCCAGTCGCCACTGCCGG. These specific primers bind to corresponding complementary regions present on two dsDNA templates used for the complex DNA mixture. These primers extend only till end of the template resulting in linearly amplified ssDNA complementary oligonucleotide sequences. The technique is quite similar to multiplex PCR with the exception that complementary ssDNA oligonucleotide fragments are generated. For purification and concentration of ssDNA fragments, ssDNA concentrator kit with purification columns from Zymo research GmbH. The 40 nt product of the MEA technique was tested for hybridization by southern blotting applying a standard protocol. Agarose and PAA-Urea gels were prepared and stained according to Sambrook and Maniatis. For all ssDNA gels, 20–100 oligo standards from IDT technologies GmbH as reference. Fermentas cloneJET TOPO cloning kit to clone PCR products in TOP10 cells. The unpurified PCR products from colony PCR were sent to Gatc Biotech for Sanger sequencing analysis [17]. Gatc viewer software was used to generate the Chromatogram from raw sequencing data.

## Results, Proof of Concept and Discussion

**Fig 3:**
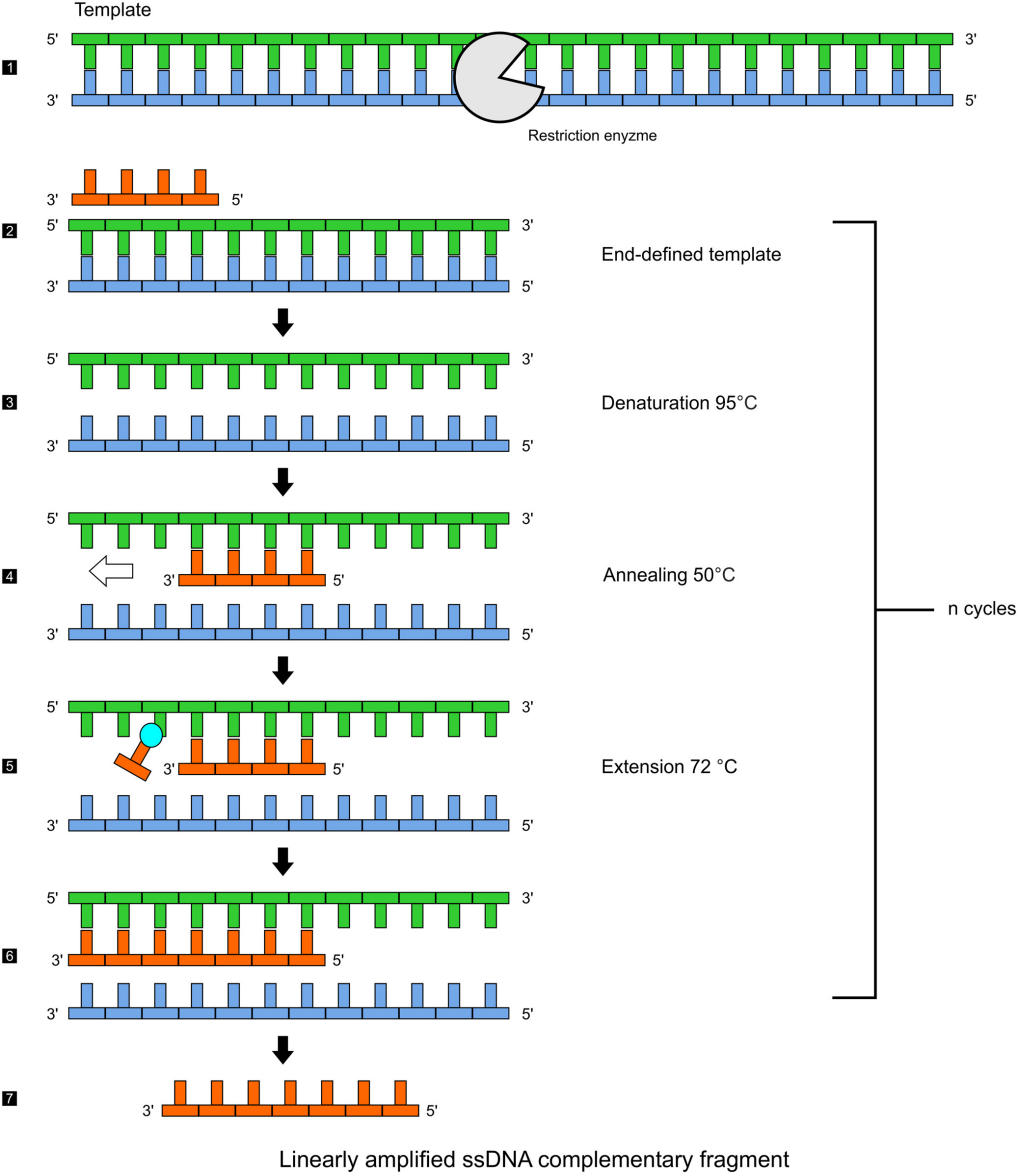
Modified end amplification (MEA) technique. The DNA template (1) requires a well defined end point of the template (2) that can be produced for instance by means of a restriction enzyme (red). Denaturation (3) at 95°C and annealing (4) at 50°C are similar to conventional PCR. Taq polymerase (blue) generates a complementary ssDNA oligonucleotide fragment from a single primer (red) during the extension step (5). The complementary ssDNA oligonucleotide fragment is extended until the end of the source strand (6). Use of a single primer results in linear amplification (7). There are n copies of complementary ssDNA oligonucleotide fragments after n cycles.

We present a novel and simple modified end amplification (MEA) technique that can generate reduced and defined amounts of data in form of short complementary oligonucleotide information fragments from a source. The technique is quite similar to multiplex PCR with the exception that complementary ssDNA oligonucleotide fragments are generated. It is based on linear amplification and generates copies of a target sequence, one copy per ssDNA molecule from one cycle. Fig 3 shows the detailed experimental procedure of the MEA technique. It differs from linear PCR techniques as following.

The single primer used in this technique produces complementary ssDNA oligonucleotide fragments, extending from the point of interest until the end of the template. The end of the template needs to be well defined such that there is no scope for further extension. This can be achieved for example with restriction enzymes. The MEA technique results in linearly amplified ssDNA sequences, which have a specific length. By modifying the primer binding region on the DNA template, the length of the linearly amplified sequences can be progressively influenced. Also, multiple primers can be used to simultaneously amplify multiple regions on either of the complementary strands of the DNA template.

Ideally, the reduced and defined amounts of oligonucleotide strands generated with the MEA technique only represent specific information. Their limited and defined length is advantageous to make hybridization highly specific.

**Fig 4:**
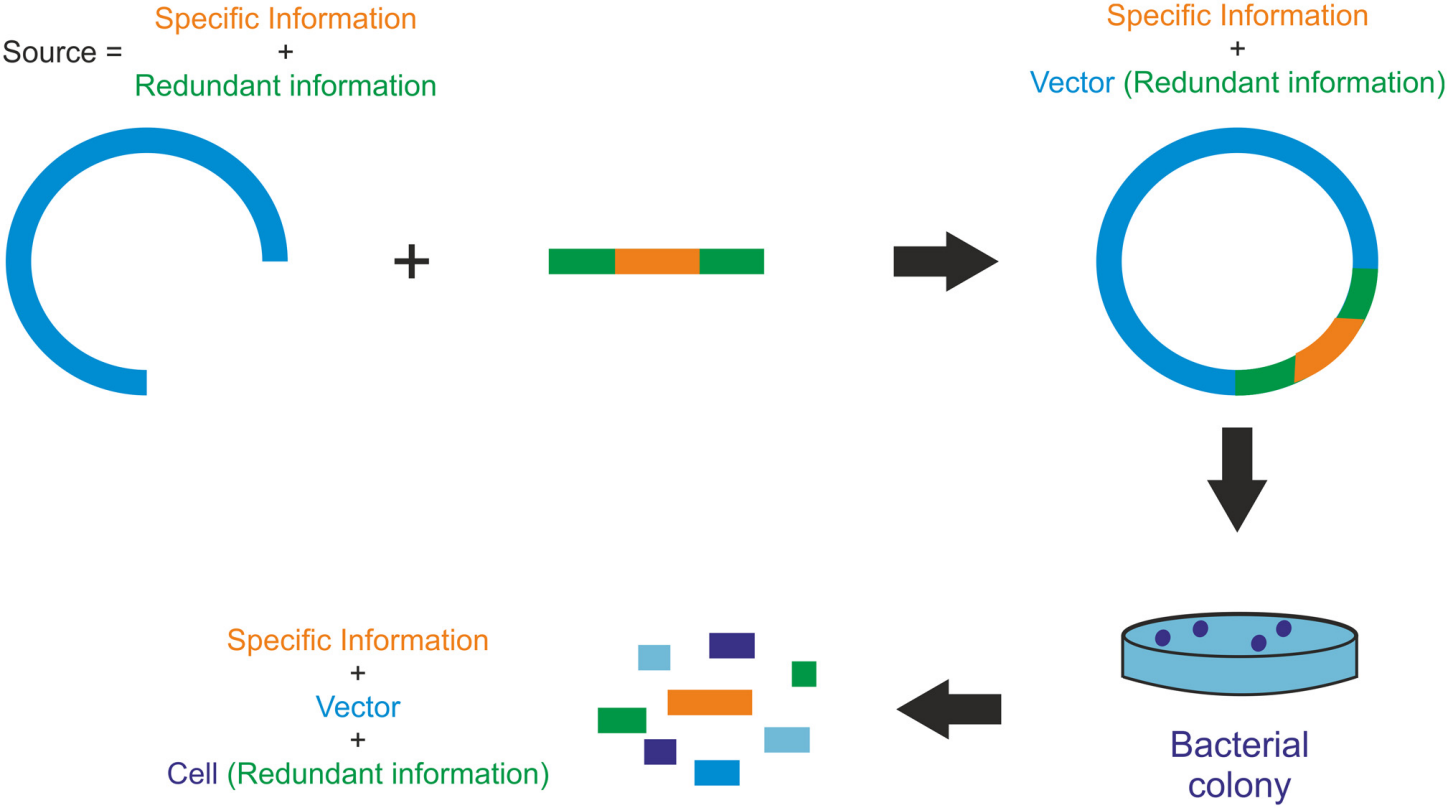
Experimental approach for development of a model system with specific information clouded by noise. Specific information of interest (orange) sandwiched by redundant information (green) is gradually clouded by vector DNA (turquoise) and cellular DNA (blue). Vector DNA and cellular DNA represent potential noise sources of increasing complexity.

As a proof of principle we show that the MEA technique can be used to successfully read, amplify and retrieve small amounts of information, even if increased amounts of competitive DNA are added to the sample. For this purpose, we produce a complex DNA mixture according to the steps in Fig 4. Through this experimental approach, the complexity of the model system can be controlled. PCR amplification and bacterial transformation result in a complex DNA mixture with specific information and redundant information. We used pJet 1.2 plasmid for cloning, which possesses high signal to noise ratio and high cloning accuracy. However the specific ssDNA target fragments (40 nt) constitute less than one percent of the cloning vector. During bacterial transformation, this cloning vector is amplified simultaneously resulting in a complex DNA mixture where the signal is hidden. The agarose gel analysis of complex DNA mixture is presented in S1 Appendix.

**Fig 5:**
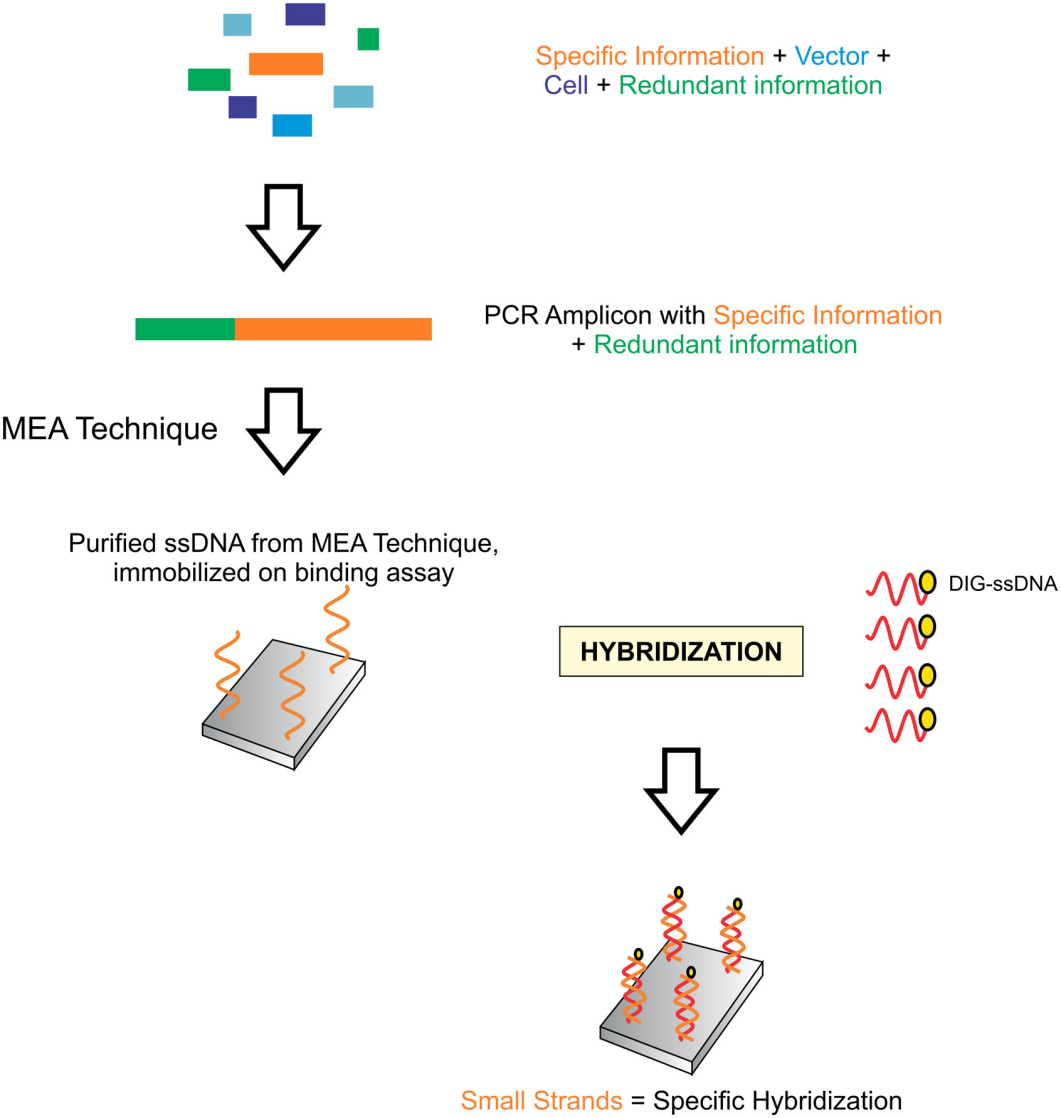
Application of modified end amplification (MEA) approach, identification, retrieval and verification of specific information. Oligonucleotide sequences are extracted with the MEA technique and purified using standard nucleic acid purification and extraction procedures. They are immobilized on a southern blot membrane by hybridizing to complementary oligonucleotide sequences with a marker. The presence of small fragments of defined length from MEA technique results in highly specific hybridization, and noise is minimized.

Fig 5 shows how our M.E.A. technique is applied to successfully retrieve specific information from a complex DNA mixture.

We verify the product of the M.E.A. technique to check for the presence of the specific information. For this purpose, the product of M.E.A. technique is extracted and purified through nucleic acid isolation and purification techniques including Biotin-Streptavidin magnetic beads procedure. The PAA-UREA gel analysis of unpurified and purified 40 nt specific strands is shown in more detail in S2 Appendix. The fidelity of the specific information is investigated with a membrane/surface based hybridization technique (Southern blotting).

**Fig 6:**
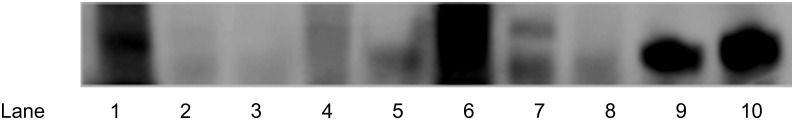
Southern blot for 40 nt sequences. The complementary sequence is labelled with a marker. This marker enables detection of the hybridized sequence through X-ray radiography. Lanes 1, 2 and 3, unpurified MEA 40 nt product in three different amounts (20, 10, 5 uL), lane 4 and 5, the ssDNA product from modified isothermal amplification method with a single primer. Lane 6–8, purified MEA products. Lanes 9–10, reference ssDNA sequences of 40 nt length, of oligonucleotide composition as the intended 40 nt sequence.

Fig 6 shows the result of the Southern blot for 40 nt sequences. The specific sequences hybridize to their complementary sequence. The specific complementary sequence is labeled with a marker. This marker renders the hybridized sequence visible through X-ray radiography for detection. In comparison with binding assays like DNA microarrays, a Southern blot requires higher concentrations of ssDNA fragments for hybridization with its complementary sequence and detection. Our findings show that the amounts of complementary ssDNA sequences generated with the M.E.A. technique could be directly used for downstream applications.

The 40 nt ssDNA sequences in lanes 1–8 represent the specific information initially embedded into the model system. Lanes 1, 2 and 3 show unpurified MEA 40 nt product in three different amounts (20, 10, 5 uL). Lane 4 and 5 show again a ssDNA product, however, from modified isothermal amplification method with a single primer instead of the MEA technique. Lane‘s 6–8 show purified MEA products. Lanes 9–10 show reference ssDNA sequences of defined length and the specific oligonucleotide sequence of the initial specific information sequence embedded in the complex DNA mixture.

The same sequences from the MEA technique, 40 nt length sequences, are cloned into longer dsDNA sequences and sequenced. The results shows that the 40 nt sequences retrieved from the model system are indeed the original information fragments embedded in the model system. The sequencing information is in S3 Appendix.

We investigate the viability of the MEA technique to retrieve multiple specific information sequences of various lengths as described in S4 Appendix. The MEA method is successfully used to identify and retrieve multiple ssDNA sequences of different lengths in a single run.

## Summary and Outlook

We have shown that a stepwise retrieval of specific information sequences from a complex DNA mixture using the M.E.A. technique can significantly reduce redundant information that usually comes along with specific information in standard microarray procedures. Also, short sequences of controlled length from the M.E.A. technique, which are below a length of 50 nt, are a better choice for specific hybridization. They exhibit fewer tendencies to form loops if compared to fragmentation techniques commonly used by commercial microarray platforms [18]. Further investigation shows that our technique can retrieve multiple specific information fragments from a source.

In some aspects, our approach compares to the SAGE technique, which generates a pool of short sequence tags from a source [19]. However, the MEA technique represents a more flexible approach that relies on a limited number of experimental steps. For future studies, a genome of higher complexity could be used instead of our model of a complex DNA mixture. Care must be taken during upscaling. It requires further investigation and validation, as higher number of primers may add another noise level. Addition of further purification steps like length based separation with reverse phase chromatography could filter the unintended ssDNA fragments and improve the signal to noise ratio.

In our study, a basic southern blot technique provides high signal to noise ratio with ssDNA target fragments generated through MEA technique from high-copy plasmids. As DNA/Genome microarrays can detect concentrations at least 1000 times below what we used for our southern blot, high-copy plasmids can be substituted by low-copy number plasmids, which generate lower concentrations of ssDNA fragments. This implies that the analytical sensitivity of our approach could be sufficient for microarray applications.

Information transmission depends on the complexity of source. Related experimental data coupled with numerical analysis could quantify effects of system complexity on signal to noise ratio that are difficult to predict with the currently available information.

Our work provides an excellent starting point for understanding information overflow in molecular screening applications. Optimal amounts of transmitted information could be determined using the MEA technique to respect the information overflow limit, which is due to the channel capacity that limits the rate of information transfer in the biomolecular communication system. Work along these lines could significantly reduce the amounts of junk data generated in affinity based molecular screening applications [20].

## Supporting Information

S1 Appendix Realization of a complex DNA mixture. (PDF)

S2 Appendix Verification and Retrieval of specific ssDNA sequences through our MEA technique. (PDF)

S3 Appendix Verification of specific information sequences with Sanger sequencing. (PDF)

S4 Appendix Multiple specific sequences of various lengths generated from MEA technique in a single run. (PDF)
