# Supplementary material for: Information Limited Oligonucleotide Amplification Assay for Affinity-Based, Parallel Detection Studies
Source: PLoS One. 2016 Mar 15;11(3):e0151072. doi: 10.1371/journal.pone.0151072 (PMC4792472; doi:10.1371/journal.pone.0151072)
Supplement: S1 Appendix — (PDF) [file pone.0151072.s001.pdf]

Information limited oligonucleotide amplification assay for affinity-based, parallel detection studies

Harish Bokkasam and Albrecht Ott

S1 Appendix

Realization of a complex DNA mixture

Model system – specific information surrounded by complex noise source. Fig. 1 shows the agarose gel analysis of the complex DNA mixture. Lane 1 shows the linearized plasmid (the vector cut with restriction enzymes). A gene replicate of 153 bp containing specific information is ligated to the linear vector and circularized. This circular vector (plasmid) is replicated as DNA in TOP 10 cells. Colony PCR is performed on the extracted plasmid to validate the presence of a 153 bp gene replicate along with specific information. The extracted plasmid containing the circular vector with specific information along with gene replicates of 153 bp length containing specific information is shown in Lane 8 without purification, Lane 9 and 10 after purification. The experimental procedure is given in detail in Figure 4 of the main text.

Lane 1: Linearized plasmid. Lane 2-6: Gene replicate/dsDNA PCR product of length 153 bp containing specific information. Lane 7-9: Gene replicates after colony PCR along with plasmid (containing specific information) after extraction and purification. Lane 10: Control PCR product 944 bp. Lane 12: Purified Plasmid-Complex DNA mixture after extraction. Lane 14-16: 1 kb ladder from New England Biolabs used as reference.

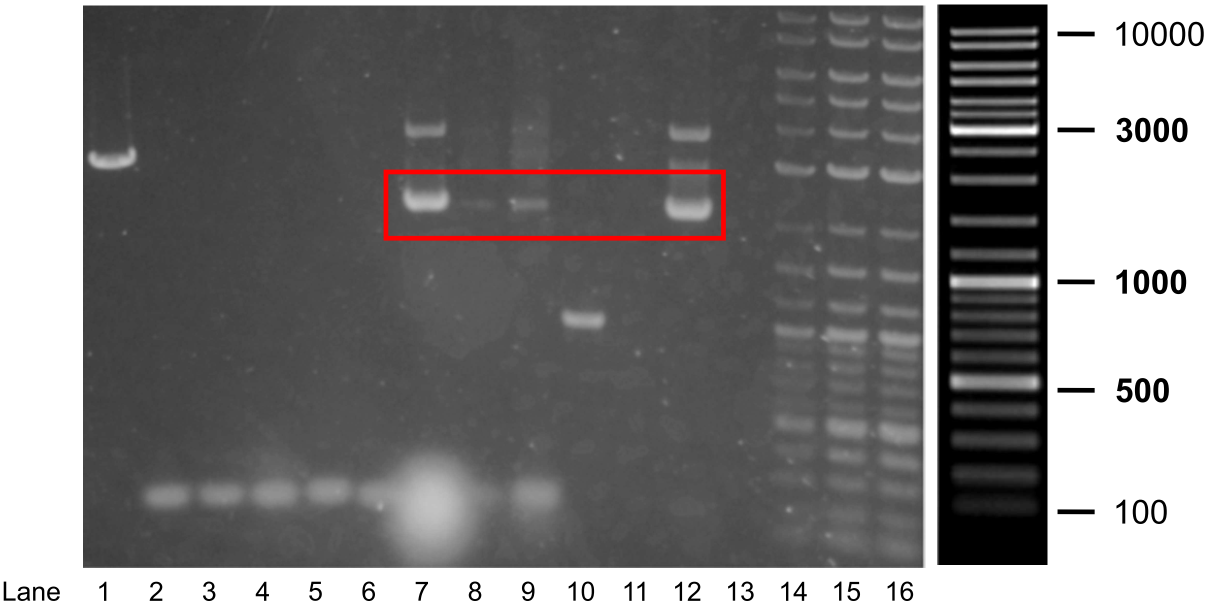

Figure 1. Agarose gel analysis of a complex DNA mixture. The generation of the complex DNA mixture follows figure 4 in the main text. The dsDNA sequence in the red box corresponds to the circular plasmid, which represents the complex DNA mixture with specific information and redundant information.
