# Supplementary material for: Information Limited Oligonucleotide Amplification Assay for Affinity-Based, Parallel Detection Studies
Source: PLoS One. 2016 Mar 15;11(3):e0151072. doi: 10.1371/journal.pone.0151072 (PMC4792472; doi:10.1371/journal.pone.0151072)
Supplement: S2 Appendix — (PDF) [file pone.0151072.s002.pdf]

Information limited oligonucleotide amplification assay for affinity-based, parallel detection studies

Harish Bokkasam and Albrecht Ott

S2 Appendix

Verification and Retrieval of specific ssDNA sequences through our MEA technique.

Fig. 1 shows a PAA-Urea 12 gel after silver staining. Lanes 3, 5 and 7 show MEA products before and after purification. After purification, most of the impurities are eliminated and the purified product is suitable for downstream applications. The length of the MEA product is 40 nt, which is of same length as the specific information embedded into the complex DNA mixture as shown in Figure 4 of main text and Appendix S1. This clearly shows that the MEA technique can produce specific information fragments of defined length.

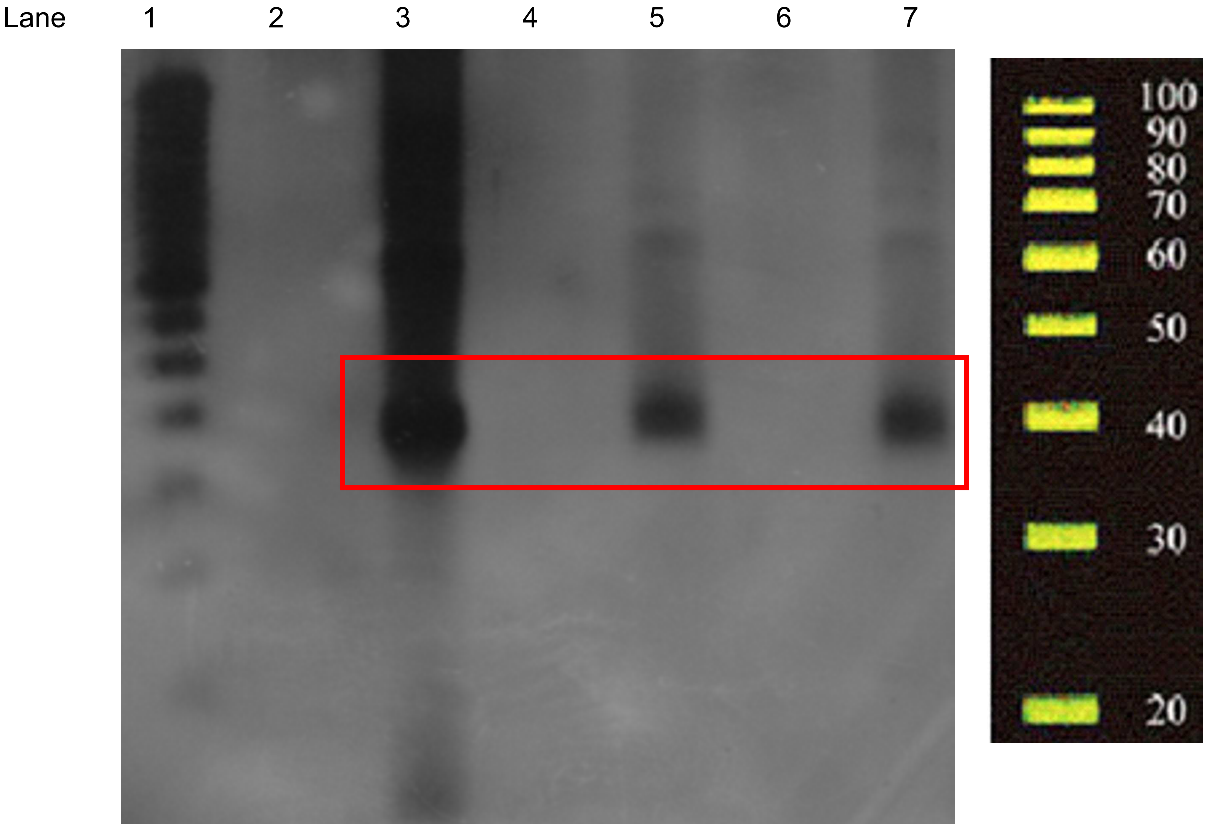

Figure 1. MEA technique products before and after purification through zymo ssDNA columns 40nt specific sequences are successfully retrieved from complex DNA mixture in a reductionist approach. Lane 1 shows a 10-100 nt ladder of ssDNA sequences. Lane 3 unpurified MEA product. Lanes 5 and 7 are purified modified PCR products with biotin streptavidin extraction and zymo ssDNA columns.
