# Supplementary material for: Information Limited Oligonucleotide Amplification Assay for Affinity-Based, Parallel Detection Studies
Source: PLoS One. 2016 Mar 15;11(3):e0151072. doi: 10.1371/journal.pone.0151072 (PMC4792472; doi:10.1371/journal.pone.0151072)
Supplement: S3 Appendix — (PDF) [file pone.0151072.s003.pdf]

Information limited oligonucleotide amplification assay for affinity-based, parallel detection studies

Harish Bokkasam and Albrecht Ott

S3 Appendix

Verification of specific information sequences with Sanger sequencing.

The various ssDNA, which were successfully extracted and purified using the MEA technique from the complex DNA mixture, are sequenced for specific oligonucleotide sequence information.For sequencing, ssDNA needs to be converted to dsDNA. The ssDNA sequence-target is hybridized with its complement in a solution based on standard hybridization protocol. Then the dsDNA is run on agarose gel electrophoresis, extracted and purified. This purified sequence is cloned into a linearized vector and transformed into E.Coli cells. Colony PCR is performed with Fermentas CloneJET kit with sequencing primers that amplify the region containing cloned hybridization products.

The resulting 135 bp sequences shown in the black box in Fig. **??** are extracted and purified from agarose gel using various commercial kits. These sequences are Sanger sequenced (Gatc Biotech) at a concentration of 15 ng/uL. The result from sequencing below reveals a fragment of 135 bp sequence containing the specific information fragment that was initially embedded into the complex DNA mixture: AT**GTTCGCGGNGGNATTCATCCATCCATTCGGTAACGCAGAT**AGGATGAATGCGGTCC

The fat letters represent the initial ssDNA sequence. This confirms the fidelity and specificity of our MEA method.

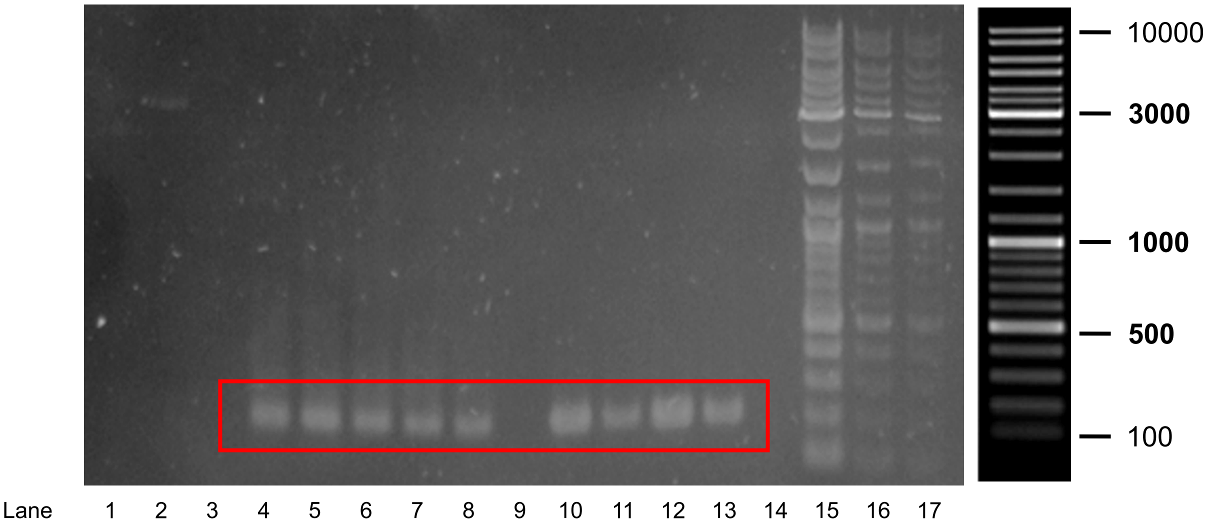

Figure 1. Colony PCR for 135 bp strands that encompass the intended ssDNA sequences The 135 bp sequences shown in the box contain the specific information of interest. The strands are sequenced after purification.
