# Supplementary material for: Information Limited Oligonucleotide Amplification Assay for Affinity-Based, Parallel Detection Studies
Source: PLoS One. 2016 Mar 15;11(3):e0151072. doi: 10.1371/journal.pone.0151072 (PMC4792472; doi:10.1371/journal.pone.0151072)
Supplement: S4 Appendix — (PDF) [file pone.0151072.s004.pdf]

Information limited oligonucleotide amplification assay for affinity-based, parallel detection studies

Harish Bokkasam and Albrecht Ott

S4 Appendix

Multiple specific sequences of various lengths generated from MEA technique in a single run.

Fig. 1 shows the specific sequences of various lengths produced in a single run through MEA technique. Lanes 1, 2 and 3 show MEA products of lengths 30, 40 and 50 nt respectively. Lane 5 and 6 show a reference ssDNA ladder of 20-100 nt. Oligonucleotide fragments of different length can be generated with MEA technique in a single experimental run.

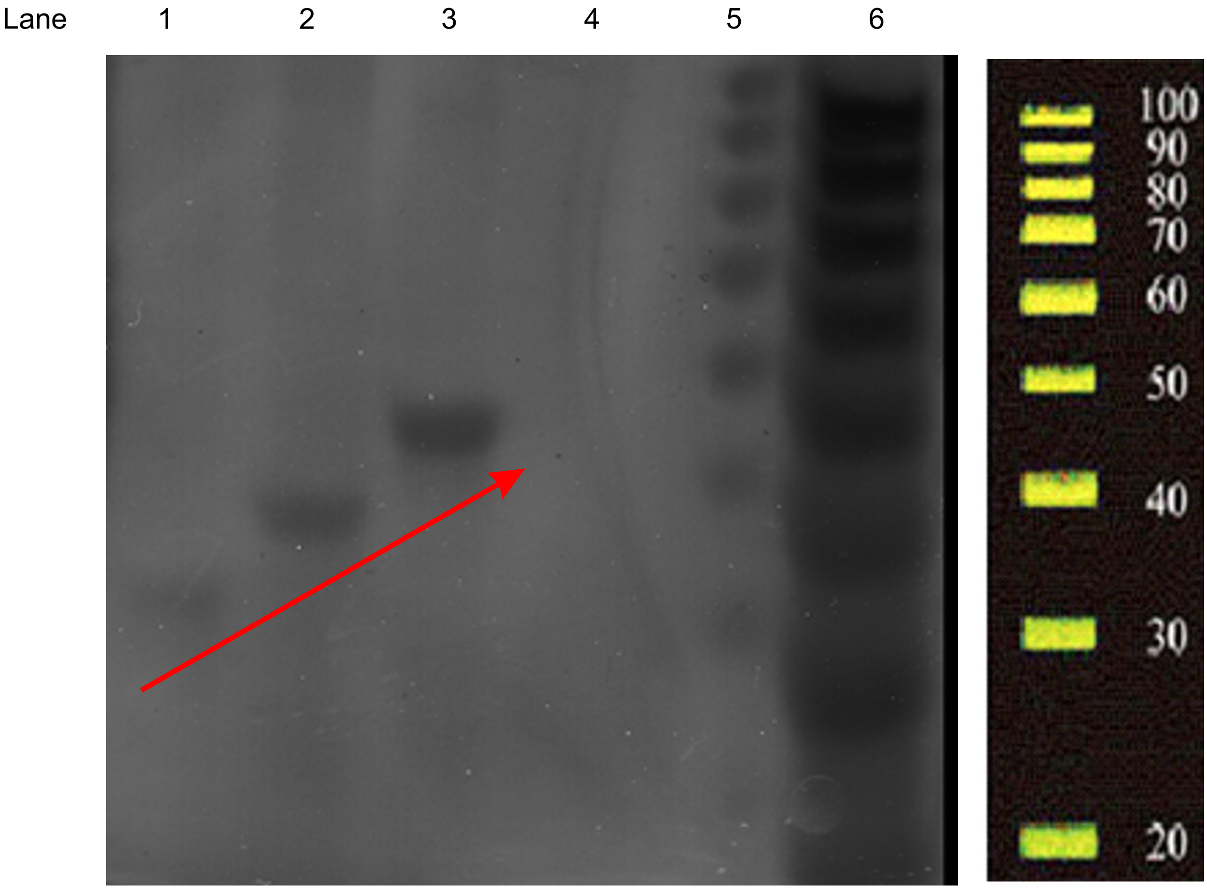

Figure 1. Multiple specific ssDNA sequences of various lengths from 30 nt to 50 nt from a complex DNA mixture. The sequences of various lengths are specific ssDNA oligonucleotide sequences generated from a complex DNA mixture through the MEA technique in a single experiment. The desired length of the specific sequences was varied by using primers which bind to their corresponding complementary sequences present in the M.E.A. template.
